# Unbalancing the Attentional Priority Map *via* Gaze-Contingent Displays Induces Neglect-Like Visual Exploration

**DOI:** 10.3389/fnhum.2020.00041

**Published:** 2020-02-20

**Authors:** Björn Machner, Marie C. Lencer, Lisa Möller, Janina von der Gablentz, Wolfgang Heide, Christoph Helmchen, Andreas Sprenger

**Affiliations:** ^1^Department of Neurology, University of Lübeck, Lübeck, Germany; ^2^Department of Neurology, General Hospital Celle, Celle, Germany; ^3^Department of Psychology II, University of Lübeck, Lübeck, Germany

**Keywords:** spatial attention, neglect, eye movements, visual exploration, gaze contingent display

## Abstract

Selective spatial attention is a crucial cognitive process that guides us to the behaviorally relevant objects in a complex visual world by using exploratory eye movements. The spatial location of objects, their (bottom-up) saliency and (top-down) relevance is assumed to be encoded in one “attentional priority map” in the brain, using different egocentric (eye-, head- and trunk-centered) spatial reference frames. In patients with hemispatial neglect, this map is supposed to be imbalanced, leading to a spatially biased exploration of the visual environment. As a proof of concept, we altered the visual saliency (and thereby attentional priority) of objects in a naturalistic scene along a left-right spatial gradient and investigated whether this can induce a bias in the exploratory eye movements of healthy humans (*n* = 28; all right-handed; mean age: 23 years, range 19–48). We developed a computerized mask, using high-end “gaze-contingent display (GCD)” technology, that immediately and continuously reduced the saliency of objects on the left—“left” with respect to the head (body-centered) and the current position on the retina (eye-centered). In both experimental conditions, task-free viewing and goal-driven visual search, this modification induced a mild but significant bias in visual exploration similar to hemispatial neglect. Accordingly, global eye movement parameters changed (reduced number and increased duration of fixations) and the spatial distribution of fixations indicated an attentional bias towards the right (rightward shift of first orienting, fixations favoring the scene’s outmost right over left). Our results support the concept of an attentional priority map in the brain as an interface between perception and behavior and as one pathophysiological ground of hemispatial neglect.

## Introduction

Selective visuospatial attention is a crucial cognitive process that enables us to detect the behaviorally relevant object(s) among all the other objects in a complex visual world (Desimone and Duncan, 1995; Fecteau and Munoz, 2006; Moore and Zirnsak, 2017). Whether an object is selected/attended, mainly depends on two factors: (i) its *salience*, i.e., the physical bottom-up distinctiveness of the object in contrast to its surrounding (Itti and Koch, 2000, 2001); and (ii) its *relevance*, defined by top-down influences such as the internal goal of the observer or external task demands (Egeth and Yantis, 1997; Einhäuser et al., 2008). Only the object with the highest “attentional priority,” a combination of salience and relevance, will be selected in a winner-takes-all fashion (Itti and Koch, 2001; Fecteau and Munoz, 2006; Serences and Yantis, 2007; Bisley and Goldberg, 2010). The neuronal basis of this process has been related to an “attentional priority map” in the brain of both monkeys and humans (Gottlieb et al., 1998; Pouget and Driver, 2000; Serences and Yantis, 2006; Bisley and Goldberg, 2010). The favored candidate locus is the parietal lobe containing multisensory neurons able to integrate stimulus-driven (bottom-up) and goal-driven (top-down) information into one topographical map of objects in space (Goldberg et al., 2006; Ipata et al., 2009; Bisley and Goldberg, 2010; Ptak and Fellrath, 2013). More specifically, there is evidence from fMRI studies (Jerde and Curtis, 2013) that an area in the posterior intraparietal sulcus and an area in the superior precentral sulcus are the most probable candidates for priority maps of space in human cerebral cortex and are proposed to be the human homologs of monkeys’ frontal eye field and lateral intraparietal area.

Direct structural damage to the parietal lobe, or collateral functional disturbance, may cause an imbalance in the attentional priority map, resulting in a biased deployment of attention and exploratory behavior in space (Pouget and Driver, 2000; Corbetta and Shulman, 2011; Ptak and Fellrath, 2013). This may lead to severe impairments in everyday life as observed in patients with hemispatial neglect following an acute unilateral (mostly right hemisphere) stroke (Parton et al., 2004; Ringman et al., 2004). The patients exhibit a lack or loss of awareness for objects, people and own body parts in the (usually left) side of space opposite to their brain lesion (Robertson and Halligan, 1999; Heilman et al., 2000). Although neglect is known to represent a multi-component syndrome, that also includes non-lateralized deficits such as an impairment in spatial working memory and sustained attention (Husain and Rorden, 2003), the egocentric bias away from contralesional and towards ipsilesional hemispace is regarded as the core symptom of spatial neglect (Parton et al., 2004; Corbetta and Shulman, 2011). This bias can be elegantly studied by using eye movement recordings during computerized tasks of spatial attention (Chédru et al., 1973; Behrmann et al., 1997; Sprenger et al., 2002; Mort and Kennard, 2003; Bonato, 2012; Machner et al., 2012, 2018).

The origin of the ipsilesional bias in neglect patients has been discussed to be a consequence of a distorted neural representation of space in the brain (Milner and Harvey, 1995; Bisiach et al., 1996), a disturbed transformation of sensory input signals into non-retinotopic spatial maps (Karnath, 1997) or a disorder of directing spatial attention (Posner et al., 1984; Kinsbourne, 1993; Corbetta and Shulman, 2011; Ptak and Fellrath, 2013). According to the attentional hypothesis, the unilateral brain damage causes an imbalance in the attentional priority map leading to a lateralization of selective attention, i.e., objects in ipsilesional (right) hemispace have a competitive advantage and are always more likely to be attended than contralesional (left) objects (Pouget and Driver, 2000; Bays et al., 2010; Ptak and Fellrath, 2013). Whether an object is located on the “left” or the “right” side, however, depends on the spatial reference frame. While the existence of a purely object-based, “allocentric,” observer-independent coordinate system is doubtful (Driver and Pouget, 2000; Filimon, 2015), the brain certainly uses various egocentric reference frames when creating a representational map of objects in space (Andersen et al., 1985; Colby and Goldberg, 1999; Pouget and Driver, 2000). Thus, neurons in the parietal lobe are able to encode the location of an object in relation to the trunk (taking the body’s midsagittal plane as the 0 meridian), the head (taking the nose as the center of an extrapolated straight ahead) and the eye position (with the fovea as the center of a retinotopic coordinate system). This concept of multiple spatial reference frames is also consistent with the notion that the awareness/unawareness for objects in space is depending on (and can be modulated by) the relative trunk, head and eye position in neglect patients (Karnath et al., 1993; Schindler and Kerkhoff, 1997; Behrmann et al., 2002).

The aim of the current study was to provide a proof of concept that an external alteration of the attentional priority map in healthy subjects can induce a neglect-like visuospatial exploration behavior. We applied gaze-contingent display (GCD) technology (Dorr et al., 2004; Machner et al., 2009) to continuously alter the saliency of objects in relation to their current location in space with respect to the observer’s head and eye position. We hypothesized that an external modification of the objects’ saliency following a spatial left-right gradient resembles the biased internal representation of objects in an imbalanced attentional priority map, as it has been proposed for patients with hemispatial neglect. Consequently, we expected our lateralized sensory modification to induce a “neglect-like” oculomotor behavior in healthy subjects, i.e., a spatially biased exploration of a visual scene reflected by exploratory eye movements. Since the “attentional priority map” is proposed to also integrate top-down influences in the selective process, our modification should alter not only saliency-driven (bottom-up) visual exploration but also goal-driven visual search. A positive finding of our virtual disease model would strengthen the pathophysiological concept of spatial neglect as a consequence of an imbalanced attentional priority map in the lesioned brain.

## Materials and Methods

### Participants

The study has been approved by the local Ethics Committee of the University of Lübeck (AZ 14-189). Written informed consent according to the Declaration of Helsinki was obtained from all participants. We recruited 28 healthy participants (23 females; mean age: 23 years, range 19–48), most of whom were students or employees at the University of Lübeck. They were all right-handed as tested by the Edinburgh Handedness Inventory (Oldfield, 1971). Participants were included if they had a visual acuity of above 0.7 and intact color vision as tested by the Ishihara’s Test (Kanehara and Company Limited, Tokyo/Japan). None of the participants had a known neurological, psychiatric or ophthalmological disease.

### Experimental Setup

#### Stimuli and Apparatus (Including Eye-Tracking and Gaze-Contingent Display Technology)

Stimuli were 10 different scenic images taken from the “Can you see what I see?” books by Walter Wick (“Dream Machine” 2003 and “Cool Collection” 2004, Scholastic Inc., New York, NY, USA). The pictures were chosen because of their complex composition of various naturalistic objects equally distributed over the whole visual scene.

The images were presented at a size of 1,920 × 1,080 pixels on a 27″ widescreen TFT monitor (BenQ XL2720, with a resolution of 1,920 × 1,080 pixels and a refresh rate of 120 Hz). At an eye-to-screen distance of 65 cm, the display covered a visual field of 50° width and 30° height.

Eye movements were recorded with a video-based eye tracker (EyeLink 1,000 Plus, SR Research Limited, Ontario, CA, USA) at a sampling rate of 1000 Hz. Head movements were minimized by the use of a chin rest.

GCD technology with the high spatial and temporal resolution was used to generate the gaze-contingent, eye-centered mask (see next section for specifications of the mask). Therefore, the current gaze position on the screen was detected by the video-based eye tracker with a spatial accuracy of 0.4° and a temporal resolution of 1,000 Hz. Using fast algorithms programmed in MATLAB^®^ with the parallel programming toolbox, the mask was superimposed on to the original image, always centered on the current gaze position and thereby “moving with the eyes.” Taking all system delays into account, the screen refresh after an eye movement occurred at least after the next monitor cycle, i.e., in less than 8 ms. This rapid screen update prevented participants from perceiving stimuli in their original salience before the mask reached the area with the new saccade.

#### Modification of the Images’ Saliency by Use of Body- and Eye-Centered Masks

**Figure 1:**
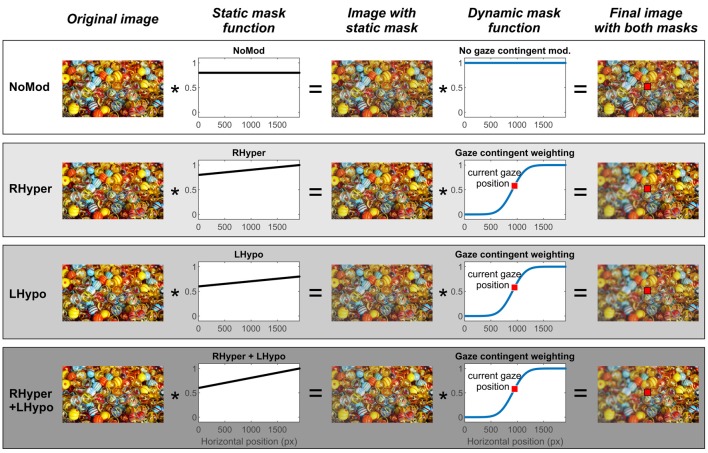
Stimulus image and the different types of modification with static (body-centered) and dynamic (eye-centered) masking. The figure depicts a schematic illustration of one exemplary original stimulus image and the individual steps of the four different modifications up to the final image presented in each modification condition. Due to copyright, we replaced the original “Can you see what I see” picture in the figure with a comparable picture downloaded from the free-to-use website https://pixabay.com/de/ (“marbles-1659398”).

We modified the saliency of the original images by superimposing different computerized masks (Figure 1). These masks were either static, i.e., stable on the screen with regards to the viewer’s fixed head and trunk position (“body-centered”), or dynamic, i.e., they constantly changed in dependence of the current gaze position (“eye-centered”). The latter was achieved by the use of the GCD technology, as described above.

There were four different modification conditions: one baseline condition without any lateralized modification (NoMod) and three lateralized modification conditions with an increasing degree of imbalance in the saliency of objects in the left and right hemispace (RHyper, LHypo, and RHyper+LHypo).

In the NoMod condition, the static mask led to a uniform 80% reduction of the image’s color saturation (i.e., without a side difference) and there was no additional gaze-contingent manipulation. In the three lateralized modification conditions, an increasing weighting function of the static mask (RHyper < LHypo < RHyper+LHypo) led to an increasing left-right imbalance of the image’s saliency with respect to the observer’s fixed head and trunk. Next, onto this “spatiotopically” modified image, a gaze-contingent (eye-centered, “retinotopic”) mask was superimposed which induced an additional “blur” on the left of the current fixation by decreasing the local contrast. This “blurring” mask was programmed using the “imgaussfilt” function within MATLAB^®^ (Sigma 10 pixels, filter size 51) and it was centered on the current gaze position with an offset of 39 pixels (~1°) and a sigma of 195 pixels (~5°).

The Supplementary Video S1 demonstrates how the combination of the lateralized static and dynamic mask (RHyper+LHypo modification condition) changed the stimulus image’s saliency (original “Can you see what I see?” image replaced due to copyright) during an exemplary scan path of one participant, his current gaze position being indicated by a red square.

#### Task Instructions to Elicit Stimulus-Driven Free Viewing and Goal-Directed Visual Search

As described above, we modified the saliency of the images by use of masks that locally changed the color saturation and contrast of objects in dependence of their spatial localization in the scene and with relation to the observer’s current gaze position.

In order to investigate the impact of this salience modification on both types of visual exploration, i.e., stimulus-driven free viewing and goal-driven visual search, we introduced two different task instructions.

In the first part of the experiment, the free viewing (FV) block, participants were asked to attentively explore the scenes without any specific task given.

In the second part of the experiment, the visual search (VS) block, an individual instruction was presented before each stimulus image that told the participant which object to look for (e.g., “a die”). The predefined target object was singular in the image and could be located within one of four virtual columns with respect to its x-position on the screen (outer left OL, center left CL, center right CR, outer right OR). Overall trials, the targets were present in the left or right hemifield with an equal probability (30% OL, 20% CL, 20% CR, 30% OR).

#### Procedure

Participants were seated in a dim-lit room in front of the stimulus screen, their head stabilized on a chin rest with an eye-to-screen distance of 65 cm and the nose aligned with the horizontal center of the screen. After a 13-point calibration of the eye tracker, the experiment started with the “free viewing” block. Each picture was presented for a duration of 20 s, meanwhile, the exploratory eye movements were recorded. There were 40 trials in the FV block, i.e., each of the 10 original images were randomly presented in all four different modifications, but the random order was kept the same in all participants.

After a short break, the participants were introduced to the “visual search” block by performing one practice trial. The target object was first named in white letters on a black background of the screen and subsequently, the stimulus picture appeared. The participant was instructed to search for the predefined target and to press a response button on a keyboard as soon as the target was detected. The stimulus picture then disappeared and the new search instruction for the upcoming picture appeared on the screen. In the VS block, pictures were presented until the target button was pressed or until the maximum time limit of 30 s run out. Again, there were 40 trials in the FV block due to the four different modification conditions of the 10 original images.

### Statistics

Statistical analyses were performed with SPSS (22.0.0.2; IBM Corporation, Somer, NY, USA). Data are reported as Mean ± Standard Error of the Mean (SEM), error bars in the figures analogously show the SEM. The FV and VS blocks were separately analyzed. In order to assess the differential influence of the four salience modifications (NoMod, RHyper, LHypo, LHypo+RHyper) on the different eye movement parameters, we performed univariate ANOVAs with repeated measures using MODIFICATION as to the within-subject factor. In some comparisons, the sphericity requirement was violated. Therefore, we report *F*-values with Greenhouse–Geisser correction but report degrees of freedom *(df)* uncorrected in order to show the factorial analysis design. In case of significant main effects, *post hoc* *t*-tests were performed and *t*-test results were shown as significant when they attained a statistical level of *p* < 0.05 after the Bonferroni correction for multiple testing was applied.

## Results

### Global Parameters of Eye Movements

We first analyzed the global eye movement parameters independent of their direction or spatial position on the screen. We calculated the total number of fixations, the mean duration of the single fixations and the saccadic amplitude. The mean values are presented in Table 1 in dependence of the type of salience modification, separately for the FV and the VS condition.

**Table 1:** Influence of saliency modification on eye movement parameters in the free viewing and the visual search condition.

	Free viewing				Visual search			
Parameters	*NoMod*	*RHyper*	*LHypo*	*RHyper+LHypo*	*NoMod*	*RHyper*	*LHypo*	*RHyper+LHypo*
*Global parameters*								
Number of fixations	65 ± 2	61 ± 2*	61 ± 2*	61 ± 2*	56 ± 3	69 ± 3*	52 ± 3	56 ± 2
Fixation duration (ms)	237 ± 5	253 ± 5*	256 ± 7*	253 ± 5*	213 ± 4	218 ± 4	220 ± 4*	218 ± 4
Saccadic amplitude (°)	3.6 ± 0.2	3.6 ± 0.1	3.9 ± 0.2	3.9 ± 0.1	4.1 ± 0.1	4.0 ± 0.1	4.4 ± 0.2*	3.9 ± 0.1
*Spatial distribution of fixations*							
The direction of first orienting	31 ± 4	53 ± 5*	58 ± 5*	62 ± 5*	32 ± 5	51 ± 6*	58 ± 5*	58 ± 5*
(% of trials started on the right)
The bias of first orienting (°)^§^	−2.3 ± 0.4	−0.7 ± 0.4*	0.3 ± 0.4*	0.4 ± 0.4*	−1.8 ± 0.5	−0.2 ± 0.6*	0.5 ± 0.6*	0.9 ± 0.6*
Center of fixation (°)^§^	−1.5 ± 0.4	−0.8 ± 0.6	−0.8 ± 0.5	−0.5 ± 0.6	−1.0 ± 0.5	−0.5 ± 0.4	−0.7 ± 0.5	−0.3 ± 0.5
Laterality index^§^	0.19 ± 0.06	0.29 ± 0.07	0.36 ± 0.07*	0.40 ± 0.07*	0.0 ± 0.05	0.15 ± 0.04*	0.24 ± 0.07*	0.17 ± 0.05*
Refixations (%)	27.3 ± 1.0	28.6 ± 1.2	27.2 ± 1.2	27.7 ± 1.3	15.1 ± 1.1	18.3 ± 0.9	15.4 ± 0.9	15.3 ± 0.7

Data are presented as mean ± SEM. *Values significantly different from the NoMod baseline condition (*p* < 0.05). ^§^Negative values indicate a bias towards the left, positive values towards the right hemifield (range −25 to +25°).

#### Number of Fixations

The ANOVA on the *number of fixations* revealed that MODIFICATION had a main effect in both the FV (*F*_(3,27)_ = 16.4, *p* < 0.001) and the VS condition (*F*_(3,27)_ = 12.5, *p* < 0.001). In FV, all three types of modifications led to a significant reduction of the overall number of fixations as compared to the NoMod condition (*p* < 0.001). In VS, only the RHyper modification had a significant impact, it increased the number of fixations as compared to the NoMod condition (*d* = 13.3 ± 3.0, *p* = 0.001). Notably, this value is confounded by the individual difficulty of the search targets because the number of fixations increased with increasing search duration until the target response button was pressed.

#### Fixation Duration

The ANOVA on the *mean fixation duration* revealed that MODIFICATION had a main effect in both the FV (*F*_(3,27)_ = 14.6, *p* < 0.001) and the VS condition (*F*_(3,27)_ = 4.9, *p* < 0.01). In FV, all three lateralized modifications led to a significant increase in the mean fixation duration as compared to the NoMod condition (*p* < 0.001). In VS, only the LHypo modification led to a significant increase as compared to the NoMod condition (*d* = 7.6 ± 2.5 ms, *p* = 0.03).

#### Saccadic Amplitude

The ANOVA on the *mean saccadic amplitude* showed a main effect for MODIFICATION in the FV (*F*_(3,27)_ = 0.49, *p* < 0.01) and the VS condition (*F*_(3,27)_ = 9.1, *p* < 0.001). However, *post hoc* *t*-tests showed that in the FV condition this was not due to a difference between the NoMod and modification conditions but due to a small difference between the RHyper and the RHyper+LHypo modification (*d* = 0.22 ± 0.07°, *p* = 0.02). In the VS condition, only the LHypo modification differed significantly from the NoMod by showing a slight increase of the saccadic amplitude (*d* = 0.28 ± 0.09°, *p* = 0.03).

### Spatial Distribution of Eye Movements

#### First Orienting

For information on the first orienting, we investigated the *direction* of the first gaze in each trial and the absolute distance between its landing position and the screen’s center (*bias*). The rationale was that healthy subjects are known to usually start their visual exploration in the left hemifield (Gigliotta et al., 2017), whereas patients with left hemispatial neglect immediately orient towards the right hemifield (Gainotti et al., 1991). The *direction of first orienting* was determined by calculating the relative number of trials started in the right hemifield (% of all trials) and the *bias of first orienting* was assessed by analyzing the × position (°) of the first gaze in every trial on the screen.

The mean results are given in Table 1. The ANOVA on the *direction of first orienting* revealed that MODIFICATION had a main effect in both the FV (*F*_(3,27)_ = 25.5, *p* < 0.001) and the VS condition (*F*_(3,27)_ = 20.3, *p* < 0.001). In both task conditions, all three modifications led to a significant increase in trials that were started in the right hemifield as compared to the NoMod (*p* < 0.001).

**Figure 2:**
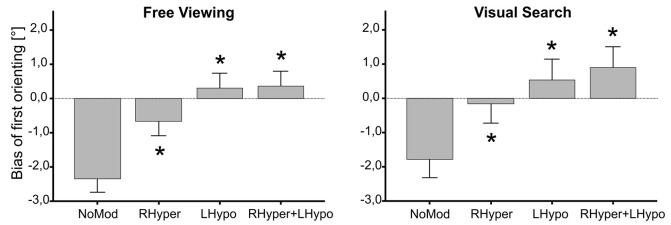
Bias of first orienting depending on the type of modification. The median × position of the first gaze samples on the stimulus screen is depicted as the “Bias of first orienting [°]” in dependence of the four different types of modification, separately for the two task conditions (free viewing, visual search). In both tasks, the three lateralized saliency modifications (RHyper, LHypo, and RHyper+LHypo) led to a significant rightward shift of the first orienting bias as compared to the NoMod condition (*p < 0.05).

The ANOVA on the *bias of first orienting* revealed that MODIFICATION had a main effect in both the FV (*F*_(3,27)_ = 20.1, *p* < 0.001) and the VS condition (*F*_(3,27)_ = 18.2, *p* < 0.001). In both task conditions (Figure 2), all three types of modification led to a significant increase in the bias, i.e., a rightward shift as compared to NoMod (*p* < 0.01).

#### Horizontal Fixation Distribution

**Figure 3:**
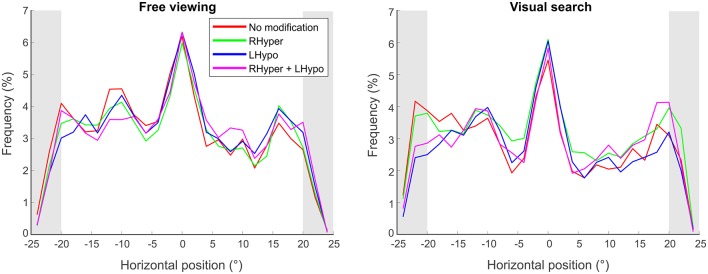
Horizontal fixation distribution. The spatial distribution of fixations is illustrated as a mean function of fixation frequency in dependence of the horizontal x-position on the screen (°), separately for the two task conditions (free viewing and visual search) and the four types of modification. Fixations landing in the outer 5° of the stimulus screen (gray area) were used for the laterality index analysis.

We analyzed the *horizontal fixation distribution* by calculating the relative number (frequency) of gaze points within 2° bins on the horizontal x-axis of the screen. Figure 3 depicts the mean results over all trials for the four different saliency modifications, separately for the two task conditions.

As shown in Figure 3 for both tasks, the bell-shaped spatial distribution of the fixations and their “peak of fixation” at the screen’s center appeared almost alike in the NoMod and the three modified conditions. Accordingly, the ANOVA on the *center of fixation*, i.e., the median × position on the screen where 50% of all fixations were located on the left and 50% on the right, revealed no main effect for MODIFICATION in both the FV (*F*_(3,27)_ = 1.1, *p* = 0.34) and the VS (*F*_(3,27)_ = 0.5, *p* = 0.65) condition. The absolute values of the center of fixation are provided in Table 1. Next, we analyzed the center of fixation as a function of time in order to look for a potential time-dependent effect of the modification and furthermore, we explored the fixation frequency of the outmost left and right part of the visual scene, in order to look for more subtle signs of biased visual exploration.

#### Center of Fixation in Dependence of Time

**Figure 4:**
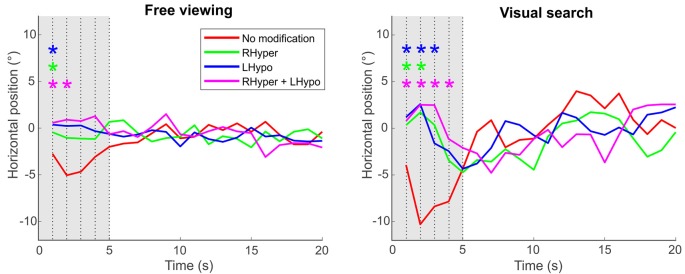
Center of fixation over the trial’s time. The center of fixation is depicted as the median horizontal gaze position on the screen (y-axis) in dependence of the trial’s time (x-axis), i.e., in 1-s-bins over the 20 s of stimulus presentation duration, separately for the two task conditions and the four types of modification. Significant differences between each of the lateralized modifications (color-coded) and the NoMod baseline condition at each second of the trial’s time are marked with an * (ANOVA with *post hoc* *t*-tests, Bonferroni-corrected *p* < 0.05). Please note that the initial leftward bias (“pseudoneglect”) in the participants’ baseline condition (NoMod, red line), evident in the first 5 s (gray area) after the onset of the stimulus image, is counterbalanced by the three saliency modifications that favor exploration of the right hemispace.

The rationale to perform analyses on the *center of fixation* as a function of time was, that within the first 5 s of presentation, a complex visual scene is known to evoke a rather “perceptive scanning” behavior, which is mainly guided by bottom-up influences (such as the stimulus’ saliency) before top-down control takes over (Sprenger et al., 2013). Figure 4 illustrates for both tasks that, within the first 5 s of image presentation, participants in the baseline condition exhibited a significant leftward bias of their exploratory fixations, a behavior that was not evident during the three lateralized saliency modifications favoring the right hemispace.

#### Left-Right Difference of Fixations at the Edge of the Scene

We analyzed the *laterality index* which defines the left-right difference of exploratory fixations in the outer parts of a visual scene (Delazer et al., 2018). This ratio was calculated by subtracting the number of fixations in the area covering the outmost left 5° of the screen from the fixations in the outmost right 5°, divided by their sum. While an index of 0 indicates an equal exploration of the outmost right and left part of the visual scene, a positive index (up to the maximum value of 1) points to an avoidance of the far left and a hyper-exploration of the far-right, as it is typically observed in patients with left hemispatial neglect (Delazer et al., 2018).

The ANOVA on the *laterality index* revealed that MODIFICATION had a main effect in both task conditions, the FV (*F*_(3,27)_ = 8.5, *p* < 0.001) and the VS (*F*_(3,27)_ = 6.4, *p* = 0.001). In FV, the LHypo (*d* = 0.17 ± 0.05, *p* = 0.009) and the RHyper+LHypo (*d* = 0.21 ± 0.05, *p* = 0.003) modification led to a greater laterality index, i.e., a stronger bias towards the right, than in the NoMod. In the VS condition, a similar increase was found for all three types of modification (Table 1, *p* < 0.05).

#### Revisiting Previously Fixated Regions (“Refixations”)

Finally, we analyzed the *rate of refixations* per picture (Table 1), i.e., the relative number of fixations that landed on previously fixated regions (Machner et al., 2005). An increased number of refixations is typically found in patients with hemispatial neglect (Husain et al., 2001; Sprenger et al., 2002).

The ANOVA on the *rate of refixations* revealed no main effect for MODIFICATION in the FV condition (*F*_(3,27)_ = 1.4, *p* = 0.256) but a main effect in the VS task (*F*_(3,27)_ = 4.4, *p* = 0.011). However, the latter was only triggered by a small difference in refixations between the RHyper condition (18.3 ± 0.9%) vs. LHypo (15.4 ± 0.9%, *p* = 0.013) and RHyper+LHypo (15.3% ± 0.7, *p* = 0.002), but there was no significant difference when compared to the NoMod condition (15.1% ± 1.1, *p* = 0.060).

## Discussion

We learned from a previous study that the attentional priority map in healthy humans can be altered by reward-based learning (Chelazzi et al., 2014). We knew that neglect patients’ exploratory eye movements in visual scenes are guided by saliency features (Ptak et al., 2009; Machner et al., 2012; Fellrath and Ptak, 2015) and that the ipsilesional oculomotor bias in left hemispatial neglect can be counterbalanced by gradually decreasing the visual salience of objects in the right hemifield (Bays et al., 2010). However, to our knowledge, this is the first study showing that neglect-like visual exploration can be induced in healthy humans by altering the attentional priority map via real-time saliency modification of visual stimuli in the left hemispace, “left” with respect to the different egocentric spatial reference frames.

This modification was achieved by the use of high-end technology including very accurate eye-tracking, fast computer algorithms and a rapidly refreshing, high-resolution widescreen. Besides a static mask that permanently reduced the visual saliency of objects in the left hemifield with respect to the fixed head and trunk (body-centered), an additional dynamic mask continuously altered the saliency of objects left of the current foveal fixation (eye-centered). Despite the dynamically changing position of objects on the retina (i.e., with every new saccade), we thereby created a permanently reduced sensory input from the left hemispace. This led to a weaker representation of left-hemispace objects in the brain’s attentional priority map and entailed an enduring disadvantage during the competition for attention which made them less likely to be attended during visual exploration (Pouget and Driver, 2000; Serences and Yantis, 2007; Ptak and Fellrath, 2013).

But did this really induce a change in the visuomotor behavior of healthy subjects similar to that observed in patients with hemispatial neglect?

In fact, the saliency modification led to several neglect-like changes in both global and local eye movement parameters. Concerning global parameters, the modification induced a significant increase of the mean fixation duration, similar to the prolonged fixation durations found in patients with hemispatial neglect (Heide and Kömpf, 1998; Sprenger et al., 2002; Machner et al., 2012; Delazer et al., 2018). In the modification conditions, subjects also revealed a reduced number of fixations in the given time, as it has been described in neglect patients (Machner et al., 2012; Delazer et al., 2018). The only neglect-like disturbance of a global parameter that we did not observe in our subjects during the modification, was a reduction of the mean saccadic amplitude (Niemeier and Karnath, 2000; Müri et al., 2009).

Besides these alterations of global eye movement parameters, our artificial neglect model induced several “local” changes with regards to the spatial distribution of fixations.

First, healthy subjects usually start their visual exploration in the left hemifield, as shown for simple line bisection tasks (Bowers and Heilman, 1980; Jewell and McCourt, 2000) but also complex visual scenes (Machner et al., 2012; Gigliotta et al., 2017). This phenomenon, known as “pseudoneglect” (Bowers and Heilman, 1980), is most probably due to hemispheric asymmetries in the organization of attentional networks in the human brain (Corbetta and Shulman, 2002; Gigliotta et al., 2017). Patients with left hemispatial neglect, in contrast, show an early orientation towards the right hemifield (Gainotti et al., 1991; Behrmann et al., 1997; Azouvi et al., 2002; Müri et al., 2009). This ipsilesional orientation bias is even found in the absence of any visual input (complete darkness; Karnath and Fetter, 1995), persists at the chronic stage while other signs of neglect have remitted (Sprenger et al., 2002), and belongs to the most sensitive signs of hemispatial neglect in neuropsychological bedside tests (Azouvi et al., 2002). The saliency modification in our study induced a neglect-like change of the first orienting. In the baseline condition, participants started visual exploration in about 70% of the trials in the left hemifield and their first gaze position was clearly left of the screen’s center, reflecting the known “pseudoneglect.” In the modification conditions, most of the trials were started in the right hemifield with a concomitant rightward shift of the first gaze points. Hence, our modification abolished the very early leftward bias (“pseudoneglect”) in healthy subjects and induced a neglect-like rightward shift of the very first attentional orienting.

By analyzing the “center of fixation” as a function of time, we could further reveal that, only in the baseline but not in the modification conditions, participants kept this significant leftward bias within the first five seconds of stimulus presentation. This time period corresponds to the saliency-driven phase of attentional orienting, i.e., the rather “perceptive scanning” (Sprenger et al., 2013) of a complex visual scene before top-down control takes over (Henderson et al., 1999; Henderson, 2003). Hence, our lateralized saliency modification induced a neglect-like behavior not only at the very beginning but also during the early saliency-driven phase of attentional orienting in the visual scene.

When focusing our analysis on the outmost parts of the visual scene, another effect of the saliency modification emerged and this was even evident over the whole time range of stimulus presentation. We detected a rightward bias of exploratory fixations in the outer parts of the visual scene, most pronounced for the strongest type of modification (LHypo+RHyper) in the free viewing condition. Such a spatial asymmetry with avoidance of the far left and “hyper-exploration” of the far right is also found in patients with left hemispatial neglect (Behrmann et al., 1997; Sprenger et al., 2002; Müri et al., 2009; Delazer et al., 2018; Machner et al., 2018).

Our modification also evoked changes during “goal-driven” visual exploration as revealed by the second block of our experiment, the visual search task. To specify, despite the strong top-down control during visual search, the saliency modification could again induce a clear rightward shift of the very first orienting, suppression of the common leftward bias at the early phase of scene exploration and a preference to explore the outer right over the outer left part of the visual scene. That the process of selective attention was also disturbed during goal-driven visual search argues for a successful alteration of the attentional priority map which is known to integrate both bottom-up and top-down signals (Fecteau and Munoz, 2006).

Our findings are in accordance with a previous study that recorded eye movements while patients with left hemispatial neglect explored an abstract, non-naturalistic visual stimulus (Bays et al., 2010). The patients’ pathological spatial bias was evident during both goal-driven visual search for targets and saliency-driven saccades to “pop-out” probes. The rightward bias could be counterbalanced to a certain degree by an adaptive algorithm that reduced visual salience of objects on the right side of the stimulus image dependent on the patient’s fixation bias in the previous trials, but of note, the manipulation was stable within each trial (i.e., not continuously gaze-dependent). The authors interpreted their findings as consistent with the concept of an attentional priority map that is damaged in patients with parietal lesions and hemispatial neglect (Pouget and Driver, 2000; Bays et al., 2010). The results of our virtual disease model corroborate this concept.

Our findings further provide empirical evidence for a previously suggested computerized lesion model of spatial neglect (Pouget and Sejnowski, 2001). Their simulated lesion in a basis function model of spatial representations induced an “imbalance in the salience of stimuli that is modulated by the orientation of the body in space.” This model referred to “retinotopic receptive fields” that are gain-modulated by the eye-in-orbita position, as it has been originally shown at the single-cell level for neurons in the parietal lobe of monkeys (Andersen et al., 1985). The authors argue that damage to the parietal lobe may induce a gradient in the representation of objects in space and could explain many of the behavioral deficits in patients with hemispatial neglect (Pouget and Sejnowski, 2001).

However, an unbalanced attentional priority map, as proposed by the studies mentioned and supported by our study, cannot fully account for all the behavioral deficits that patients with the full-blown clinical picture of neglect exhibit. For instance, our lateralized saliency modification did not lead to a significant increase of “refixations,” i.e., repetitive fixations on locations that have been visited before. Those refixations are typically found in neglect patients and might reflect spatial working memory deficits and/or motor perseveration (Husain et al., 2001; Sprenger et al., 2002; Mannan et al., 2005). This underlines the heterogeneous nature of spatial neglect as a multi-component syndrome which cannot be fully explained by one pathophysiological account or lesion model (Parton et al., 2004).

Our modification could also not induce an enduring shift of exploratory eye movements towards the right hemifield as it is typically seen in patients with hemispatial neglect who exhibit an ipsilesional shift of the “center of fixation” of about 5–15° (Karnath and Fetter, 1995; Machner et al., 2012, 2018). There are different possible explanations: first, our saliency modification might not have been strong enough to elicit such a pronounced shift of the center of fixation. However, we could not further increase the physical properties of the masks (i.e., greater reduction of contrast and saturation or a complete blanking mask) nor bring the gaze-dependent mask closer to the center of foveal vision, because healthy subjects with such a “virtual hemianopic scotoma” would show a top-down guided “curiosity” with increased (and not decreased) exploratory eye movements of the masked hemifield, similar to patients with a homonymous hemianopia (Zihl, 1995; Machner et al., 2009). Thus, an increase of the saliency mask would have gone on the expense of their rather subliminal nature, which was crucial to creating a neglect-like unawareness of the deficit. We would even propose that our modification was only able to induce “neglect-like” eye movement patterns to a certain degree because it constituted a successful balancing act on the thin line between the necessary reduction of visual saliency and a subliminal nature (unawareness) of the intervention.

The second explanation refers to the different pathophysiological concepts of the ipsilesional oculomotor bias in neglect patients. While our modification basically worked via alteration of the attentional priority map, it did not influence the participants’ subjective mid in space nor directly changed the central coordinates for the representation of space. Karnath has argued that the ipsilesional shift of the center of fixation is not due to an attentional deficit but to a disturbed transformation of multimodal sensory signals into the topographic map of space in the damaged brain of neglect patients (Karnath and Fetter, 1995; Karnath, 1997). This alleged conflict between an attentional and transformational account was partly reconciled by the concept of parietal neurons integrating attention-relevant bottom-up and top-down signals into one representational map of space (Pouget and Driver, 2000). However, while our pure sensory modification could successfully simulate an attentional gradient by affecting the bottom-up signals (saliency) in different egocentric spatial reference frames, it certainly could not influence the neuronal transformation of these signals into the brain’s representational map of space nor disturb the top-down control of systematic visuospatial exploration.

## Conclusion and Perspective

This proof-of-concept study provides evidence that artificially reducing the salience of objects in the left hemispace—“left” with respect to both head/trunk- and eye-centered spatial reference frames—is able to induce a “neglect-like,” spatially biased visual exploration in healthy subjects. The finding underlines the proposal that the ipsilesional attention bias found in patients with hemispatial neglect arises from an imbalanced attentional priority map that integrates bottom-up and top-down signals into one topographic representation of objects in space by use of different egocentric spatial reference frames (Pouget and Driver, 2000; Pouget and Sejnowski, 2001). Furthermore, that such a modification of the sensory (visual) input is able to induce behavioral changes (visuospatial exploration), supports the conceptual view of the attentional priority map in the parietal lobe as an interface between perception and action (Gottlieb, 2007; Bisley and Goldberg, 2010; Ptak and Fellrath, 2013).

We imagine at least two potential future applications of our gaze-contingent approach. First, the mask could be used to better model the different sensory/attentional subcomponents of neglect. We, therefore, share our original code (see Supplementary Material Data Sheet S1) which may be refined by other researchers in the field to induce changes in exploratory eye movements of healthy subjects that get even closer to those of neglect patients. Second, the gaze-contingent approach could be applied in a modified version as a therapeutic intervention during neglect rehabilitation. Therefore, the mask would be mirrored in order to relatively increase the saliency of objects in the contralesional (left) hemifield while objects in the ipsilesional (right) hemifield would be attenuated in their attentional weight. Such an intervention during a visual exploration training in patients with hemispatial neglect could potentially counterbalance the ipsilesional attention bias and concomitant rightward shift of exploratory EM in left hemispatial neglect, which might help to reduce functional consequences and neglect-related disability.

## Data Availability Statement

The datasets generated for this study are available from the corresponding author upon reasonable request.

## Ethics Statement

The studies involving human participants were reviewed and approved by Ethics Committee of the Universität zu Lübeck. The patients/participants provided their written informed consent to participate in this study.

## Author Contributions

BM, WH, CH, and AS contributed to the conception and design of the study. ML, LM, JG, and AS organized the database. BM, ML, and AS performed the statistical analysis. BM wrote the first draft of the manuscript. LM, JG, WH, CH, and AS wrote sections of the manuscript. All authors contributed to manuscript revision, read and approved the submitted version.

## Conflict of Interest

The authors declare that the research was conducted in the absence of any commercial or financial relationships that could be construed as a potential conflict of interest.
