# Supplementary material for: Unbalancing the Attentional Priority Map via Gaze-Contingent Displays Induces Neglect-Like Visual Exploration
Source: Front Hum Neurosci. 2020 Feb 20;14:41. doi: 10.3389/fnhum.2020.00041 (PMC7045871; doi:10.3389/fnhum.2020.00041)
Supplement: Supplementary file 2 [file Data_Sheet_1.ZIP › GC_StimulusModification.html]

GC\_StimulusModification 

## Contents

- Test whether Psychtoolbox is installed
- Read example image and select modification type
- GPU usage
- Some settings
- Open Psychtoolbox screen
- Prepare calculations
- Static modification
- Nearly ready to go, wait until all buttons and keys are released
- Main loop with dynamic modification
- Finished! Clean up

```
function GC_StimulusModification(varargin)
```

```
% GC_STIMULUSMODIFICAION    Example code for gaze contingent image
%       modification from Machner et al. 2020, Frontiers Human Neuroscience
% GC_StimulusModification   calls this function, modification type
%       has to be selected by the experimenter
% GC_StimulusModification(Type)  Four types of modification are available
%       (see below), Type can be 1 to 4
%
% This function uses Psychtoolbox (http://psychtoolbox.org)
% Furthermore - if available - this function uses the Parallel Computing
% Toolbox within Matlab. If not available, the function will use
% conventional RAM and CPU(s) which is quite slow in comparison with
% graphic card computations (GPU). Therefore, the timings of the stimulus
% presentations are quite incorrect! It is recommended to use a graphic
% card with more than 500 cores, current CUDA and Matlab's Parallel
% Computing Toolbox.
%
% Copyright Andreas Sprenger, 25.January 2020
```

## Test whether Psychtoolbox is installed

```
try
    OldVerbosity = Screen('Preference', 'Verbosity', 0);
    KbName('UnifyKeyNames');
    ExitKey = KbName('ESCAPE'); % Function can be finished by ESC key or mouse button press
catch
    errordlg('Hhm, Psychtoolbox seems to be not installed. Please install Psychtoolbox first', 'Error');
    return;
end
```

## Read example image and select modification type

In this example we use a free image from pixabay.com https://pixabay.com/de/photos/murmeln-glasmurmeln-kugeln-rund-1659398

```
MImg = imread('Marbles_1920x1080.jpg');

if ~nargin
    ModificationType = menu('Select modification type', 'no modification', 'right hyper', ...
        'left hypo', 'right hyper + left hypo');
    if ModificationType == 0; return; end
else
    ModificationType = varargin{1};
end
```

## GPU usage

Fast image modification and presentation requires Matlab Parallel Computing Toolbox with a powerful graphic card (NVIDIA preferred) We try whether toolbox is installed and CUDA driver is accepted

```
try
    gpuArray((1 : 10));
    GPUflag = true;
catch
    GPUflag = false;
    disp(['Parallel Computing Toolbox not available, conventional CPU and memory will be used. ', ...
        'Presentation timings are inaccurate!'])
end
```

## Some settings

```
Param.CDFsigma   = 5;          % sigma(deg) of cdf function used to manipulate images.
Param.CDFoffset  = 1;          % offset (deg) of cdf function
Param.ScreenWidthCM = 59.5;    % BenQ XL2711; please adjust accordingly
Param.Eye2Screen = 65;         % distance eye to screen (cm)

Param.ScreenID   = 1;
Param.ScreenWidthDegree = atand((Param.ScreenWidthCM / 2) / Param.Eye2Screen) * 2;

Param.BGColor = zeros(1, 3);        % Background color (zeros = black)
Param.FGColor = ones(1, 3) * 255;   % Foreground color ([255, 255, 255] = white)
```

## Open Psychtoolbox screen

```
Screen('Preference', 'SkipSyncTests', 2);
if max(Screen('Screens')) < Param.ScreenID; Param.ScreenID = max(Screen('Screens')); end

[w, rect] = Screen('OpenWindow', Param.ScreenID, Param.BGColor);
Param.ScreenWidth = rect(3);
Param.ScreenHeight = rect(4);
Param.ScreenFrequency = Screen('GetFlipInterval', w) * 1000;
Param.PixelPerDegree = Param.ScreenWidth / Param.ScreenWidthDegree;
```

## Prepare calculations

Transfer offset and sigma degree values to pixels

```
Param.CDFoffset = Param.CDFoffset * Param.PixelPerDegree;
Param.CDFsigma  = Param.CDFsigma * Param.PixelPerDegree;

% Resize image in case of a screen with less than 1920 pix (hor.)
if Param.ScreenWidth < 1920
    MImg = imresize(MImg, Param.ScreenWidth / size(MImg, 2));
end

% We simulate eye movement data by mouse cursor position
OldMouseType = ShowCursor('CrossHair');

% Vectors for weight function
if GPUflag
    VectorHor = gpuArray((1 : size(MImg, 2))');
    OnesImgVer = gpuArray(ones(size(MImg, 1), 1));
    originalImage = gpuArray(double(MImg));
else
    VectorHor = (1 : size(MImg, 2))';
    OnesImgVer = ones(size(MImg, 1), 1);
    originalImage = double(MImg);
end
```

## Static modification

```
switch ModificationType
    case 1 % background image -> 80% gray
        if GPUflag
            Img = gather(uint8((originalImage - 128) .* ones(size(MImg)) * 0.6 + 128));
        else
            Img = uint8((originalImage - 128) .* ones(size(MImg)) * 0.6 + 128);
        end
    case 2 % gradually increase from 80% left to 100% right
        lw = (0.8 : 1/(size(MImg, 2) / 0.2) : 1);
        if length(lw) > size(MImg, 2); lw = lw(1 : size(MImg, 2)); end
        lw2 = OnesImgVer * lw;
        if GPUflag
            Img = gather(uint8((originalImage - 128) .* repmat(lw2, [1, 1, 3]) + 128));
        else
            Img = uint8((originalImage - 128) .* repmat(lw2, [1, 1, 3]) + 128);
        end
    case 3 % gradually increase from 60% left to 80% right
        lw = (0.6 : 1/(size(MImg, 2) / 0.2) : 0.8);
        if length(lw) > size(MImg, 2); lw = lw(1 : size(MImg, 2)); end
        lw2 = OnesImgVer * lw;
        if GPUflag
            Img = gather(uint8((originalImage - 128) .* repmat(lw2, [1, 1, 3]) + 128));
        else
            Img = uint8((originalImage - 128) .* repmat(lw2, [1, 1, 3]) + 128);
        end
    case 4  % gradually increase from 60% left to 100% right
        lw = (0.6 : 1/(size(MImg, 2) / 0.4) : 1);
        if length(lw) > size(MImg, 2); lw = lw(1 : size(MImg, 2)); end
        lw2 = OnesImgVer * lw;
        if GPUflag
            Img = gather(uint8((originalImage - 128) .* repmat(lw2, [1, 1, 3]) + 128));
        else
            Img = uint8((originalImage - 128) .* repmat(lw2, [1, 1, 3]) + 128);
        end
end

if ModificationType > 1
    if GPUflag
        blurredImage = gpuArray(double(imgaussfilt(originalImage, 10, 'FilterSize', 51))); % blurred image
    else
        blurredImage = double(imgaussfilt(originalImage, 10, 'FilterSize', 51)); % blurred image
    end
end

% state "originalImage" as the static modified image
if GPUflag
    originalImage = gpuArray(double(Img));
else
    originalImage = double(Img);
end
```

## Nearly ready to go, wait until all buttons and keys are released

Wait until all mouse buttons are released

```
[~, ~, buttons] = GetMouse;
while any(buttons)
    % wait until mouse button is released
   [~, ~, buttons] = GetMouse;
end
% Check whether a key is pressed
while KbCheck; end
```

## Main loop with dynamic modification

we simulate eye gaze positions by mouse position

```
LastX = Param.ScreenWidth / 2;
while 1
    % Check key presses. Exit on pushing ESC key
    [KeyIsDown, ~, KeyCode] = KbCheck;
    if KeyIsDown
        if find(KeyCode) == ExitKey; break;end
    end

    % Get mouse position and button presses. Exit on mouse button click
    [X, Y, buttons] = GetMouse;
    if any(buttons); break; end

    % Check horizontal mouse position
    if X < 0; X = X + Param.ScreenWidth; end
    if X < 0; X = 0; end
    if X > Param.ScreenWidth; X = Param.ScreenWidth; end

    if abs(LastX - X) > 5
        % With GPU computing, all modifications are computed on the graphic
        % card.
        %
        % calculate weighting function
        lw = normcdf(VectorHor, X - Param.CDFoffset(1), Param.CDFsigma(1));
        % replicate the function for the 3 dimensions of the image
        lw2 = repmat(lw, [1, size(MImg, 1), 3]);
        % permute the dimensions
        lw3 = permute(lw2, [2, 1, 3]);
        % calculate the inverse function
        lw3_inv = (lw3 - 1) * -1;   % inverse of lw3

        % multiply images with weight matrizes and sum.
        if GPUflag
            %Gather image from GPU to memory
            gcImage = gather(uint8(originalImage .* lw3 + blurredImage .* lw3_inv));
        else
            gcImage = uint8(originalImage .* lw3 + blurredImage .* lw3_inv);
        end

        % Now show the results
        % Create texture
        Htexture = Screen('MakeTexture', w, gcImage);
        % Draw texture to graphic card
        Screen('DrawTexture', w, Htexture);
        % Add current "gaze" position
        Screen('DrawDots', w, [X;Y], 10, [255, 0, 0], [], 2);
        % Show screen
        Screen('Flip', w);
        % Delete current texture from graphic card otherwise memory
        % problems will occur
        Screen('Close', Htexture);
    end
end
```

## Finished! Clean up

```
Screen('Preference', 'Verbosity', OldVerbosity);
ShowCursor(OldMouseType);
sca;
```

Published with MATLAB® R2019b
