# Supplementary figures and images for: Unbalancing the Attentional Priority Map via Gaze-Contingent Displays Induces Neglect-Like Visual Exploration
Source: Front Hum Neurosci. 2020 Feb 20;14:41. doi: 10.3389/fnhum.2020.00041 (PMC7045871; doi:10.3389/fnhum.2020.00041)

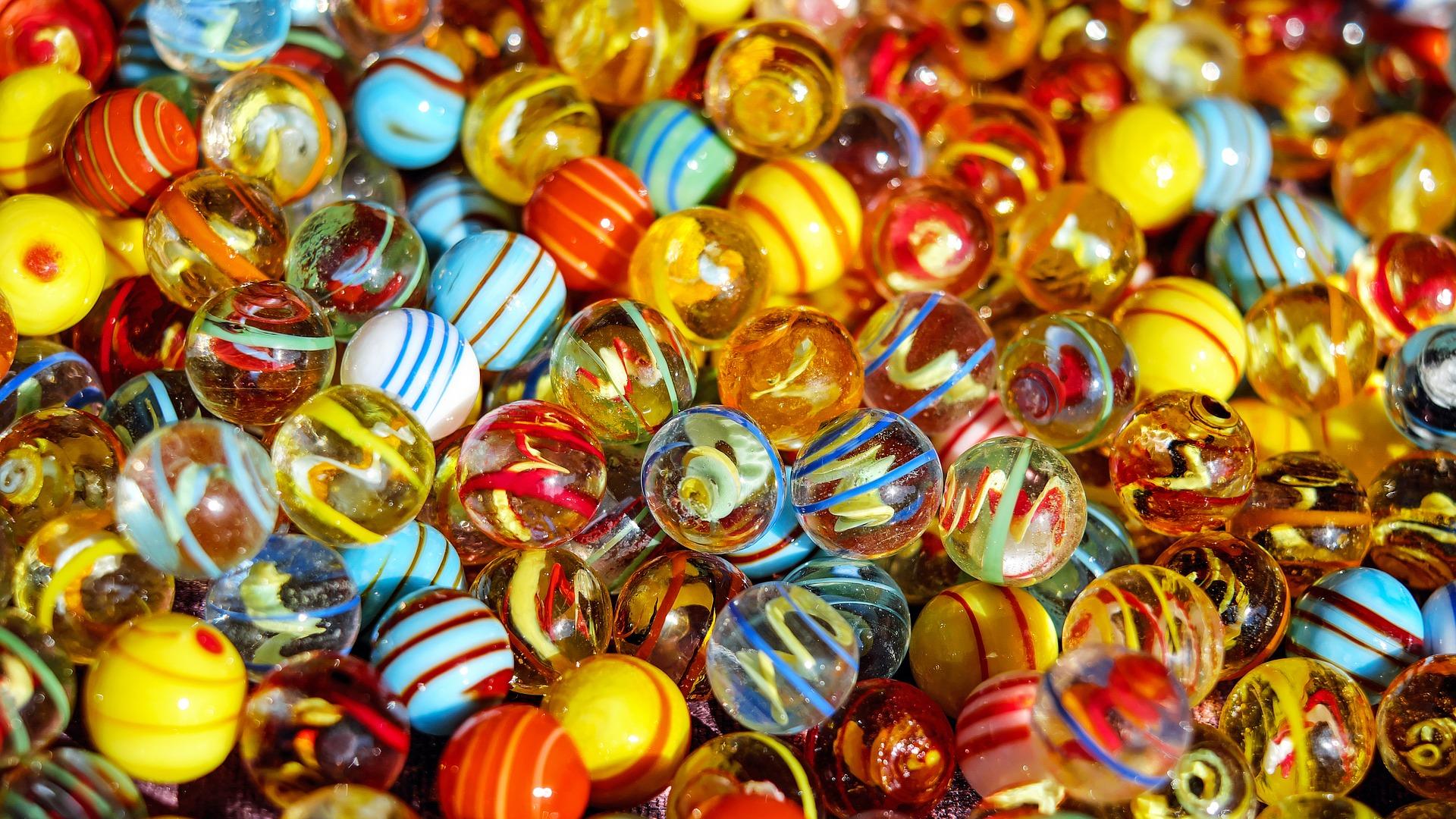

Supplement: Supplementary file 2 [file Data_Sheet_1.ZIP › Marbles_1920x1080.jpg]
